# Beyond the label: current evidence and future directions for the interrelationship between electronic cigarettes and mental health

**DOI:** 10.3389/fpsyt.2023.1134079

**Published:** 2023-08-14

**Authors:** Malia Bautista, Allison S. Mogul, Christie D. Fowler

**Affiliations:** Department of Neurobiology and Behavior, University of California, Irvine, Irvine, CA, United States

**Keywords:** nicotine, anxiety, depression, e-cigarette, vape, constituents, CYP450

## Abstract

Electronic cigarette use has dramatically increased over the last decade. With this recent technological development and wide range of constituents in various products, putative adverse effects on the brain and body have been largely unexplored. Here, we review current evidence linking electronic nicotine cigarette use with potential health consequences and provide evidence supporting an association between drug use and depression in humans. We also examine the biological effects of individual constituents in electronic cigarette aerosols, which include labeled ingredients, such as propylene glycol, vegetable glycerin, nicotine, and flavorants, as well as unlabeled ingredients found in the aerosols, such as carbonyls and heavy metals. Lastly, we examine the effects of electronic cigarette use on endogenous metabolism via changes in cytochrome P450 enzymes, which can thereby impact therapeutic outcomes. While the current evidence offers insight into the potential effects of electronic cigarette use on biological processes, further studies are necessary to determine the long-term clinical relevance of aerosol inhalation.

## Introduction

*“We don’t think a lot about addiction here […] anything about health is not on our mind”* – statement by a JUUL electronic cigarette engineer (1)

Since tobacco cigarette use continues to be a main cause of preventable death in the US and worldwide (2), significant efforts have been focused on developing smoking alternatives and therapeutics to support long-term cessation (3, 4). Initially developed as an innovative smoking cessation tool, electronic cigarettes have become increasingly popular among adult smokers, in addition to an unfortunate prevalence of use among adolescents and never-smokers (5, 6). This phenomenon may be attributed to how electronic cigarettes have evolved over time. Specifically, electronic cigarettes have become more inconspicuous, looking less like a tobacco cigarette, and their improved liquid solvents have been formulated to decrease the harshness of the smoking experience. Through a heating coil, electronic cigarettes aerosolize a liquid solvent, which is then inhaled by the user. Commercial liquids for electronic cigarettes contain nicotine and various constituents/flavorants dissolved in a vehicle solution, which is often propylene glycol and vegetable glycerin (7). However, additional constituents are also often present in electronic cigarette liquid and aerosol (7, 8), thus exposing the smoker to potentially numerous harmful substances.

In adult tobacco smokers, electronic cigarettes have been shown to be beneficial as cessation aids, but ultimately, the scientific evidence has indicated limited efficacy to reduce tobacco use over the long-term (9–14). It is important to note that electronic cigarette use among adolescents and never-smokers is increasingly prevalent, with reports indicating that use has more than doubled between 2017 and 2019 in Americans aged 16–19 (15). Commercial electronic cigarette products are continuing to evolve across time which may lead to increased intensity of use, for instance based on increasing nicotine levels, pH modifications, and potentially other design features. Importantly, electronic cigarette use has a high comorbidity with increased self-reported depression and anxiety symptoms (16, 17) Therefore, the association between electronic cigarettes and mental health will likely continue to evolve over time, especially given ease of inconspicuous use in home environments, school settings, and workplaces. It has become essential to understand how electronic cigarette use correlates with mental health, the impact on the brain’s neurobiology, and the potential impact on pharmaceutical therapies related to mental health. In this review, we examine the effects of individual constituents emitted from electronic cigarettes and the potential intersections with health. We further discuss how metabolic processes can be disrupted by constituents in electronic cigarettes, thereby impacting the effectiveness of pharmacological therapeutics used to treat mental health disorders, such as depression.

## Factors influencing electronic cigarette use

### Electronic cigarette harm perception

A multitude of factors influence the likelihood to initiate electronic cigarette use (18). Not surprisingly, both adolescent and adult electronic cigarette users are more likely to start smoking tobacco cigarettes within 2 years, as compared to non-users (19, 20). Because of this, it is especially important to understand factors that may predict the onset of electronic cigarette use in never-smokers. For instance, electronic cigarette perception was shown to be a predictor of smoking initiation amongst adolescents and adults (21, 22). Therefore, by deepening our understanding of how different sociodemographic factors and perception interrelate, vulnerable populations can be identified to prevent future initiation of drug use.

The relationship between sociodemographic factors and electronic cigarette use in adolescents is well established. In a recent survey, adolescents between 13 and 18 years of age were found to vary in their perceived harm of electronic cigarettes based on socioeconomic status, ethnic background, and parental educational attainment (23). Specifically, a lower perceived risk of electronic cigarettes was associated with a lower-income family, non-Hispanic Black ethnic background, or both parents having had no college degree (23). Furthermore, adolescents and young adult never users who were non-Hispanic Black reported a greater susceptibility to initiate disposable or reusable electronic cigarette use (24). As adolescents age, perceptions toward electronic cigarettes may persist and thus influence their likelihood of nicotine use. Not surprisingly, the internet and social media have become a powerful source of influence for both adolescents and young adults. Indeed, the advertisement of electronic cigarettes has been mainly found in online sources (25). For instance, a large number of electronic cigarette vaping-related videos are available on TikTok, a short form social media platform popular among adolescents and young adults (26). These vaping-related TikTok videos were largely found to positively depict electronic cigarette use (26). This is alarming given that advertising via social media significantly influences the perception of electronic cigarette harm in adolescents, even when controlling for exposure to warning labels and anti-tobacco advertising (27). Conversely, this highlights the potential by which social media can be used to communicate the harms of electronic cigarette use to these same adolescent and young adult populations.

Interestingly, a study in young adults found that advertising alone did not influence electronic cigarette use, but when individuals who had a low harm perception of electronic cigarettes viewed this advertising, they were then more likely to initiate electronic cigarette use (25). These findings are consistent with another study in adults, in which harm perception of electronic cigarettes in non-users could predict their status a year later (28). Specifically, increased harm perception of electronic cigarettes was significantly correlated with a lower incidence of future electronic cigarette use, whereas decreased harm perception was associated with increased likelihood of drug use (28). Given the recent regulations in over 30 states targeting electronic cigarette advertising, sales, and use (29), it will be interesting to see how electronic cigarette perceptions in adolescents and adults evolve to influence future use patterns.

### Depression and anxiety

Over 20 million adults in the United States suffer from depression (30), and multiple studies have demonstrated comorbidity between depression and electronic cigarette use (16, 31–39). This association is also dose-dependent, where greater amounts of nicotine consumed were associated with greater self-reported depressive symptoms (40). Adolescents and adults who suffer from mental illness are more likely to be both electronic and tobacco cigarette users (14, 41–44), so these populations are disproportionately burdened with health consequences associated with both types of cigarettes. Interestingly, electronic cigarette use was associated with subsequent tobacco cigarette initiation, which then correlated with depression symptoms (39), illustrating that the initial use of electronic cigarettes in never-smokers can lead to adverse health consequences. Additionally, self-report studies reveal that never smokers and smokers who have quit report overall higher levels of positive affect and lower levels of negative affect compared to current tobacco smokers (45, 46). Although prior studies provide some insight for the intersection of nicotine use and depression, it had remained unclear as to whether symptoms related to depression led to the initiation of drug use, or if the effects of drug use (e.g., alterations in brain circuitry and/or induction of withdrawal symptoms during periods of abstinence) triggered the onset of depressive symptomology. Two longitudinal studies have investigated this question regarding electronic cigarette use, yielding somewhat conflicting results (18, 47). In the first study (47), college students were surveyed at two time points within 1 year, assessing for changes in electronic cigarette use and self-reported depressive symptoms. In this sample, reported symptoms of depression predicted electronic cigarette use at both 6 month and 1 year follow-ups, whereas electronic cigarette use did not predict depressive symptoms at either time point (47). These data suggest an unidirectional relationship, in which greater depressive symptomology increases the likelihood of electronic cigarette use (47). These data also support the “self-medication” hypothesis, in which people experiencing depression may seek out substances to ameliorate their symptoms. Given that nicotine in electronic cigarettes has been shown to induce transient effects of mild euphoria, increased energy, heightened arousal and relaxation, these effects can theoretically counteract an individual’s perceived depression-associated symptoms (48). Of note, this phenomenon has also been observed in tobacco cigarette users (49, 50). Contrary to these unidirectional effects (47), an interesting longitudinal study by Leventhal and colleagues provides evidence of a bidirectional relationship between nicotine use and depression (18). This study surveyed high school students for 1 year who reported never having used any nicotine products and assessed their self-reported depression symptoms and electronic cigarette use (18). Participants with a self-reported elevation of depression-associated symptoms at baseline were more likely to use both electronic cigarettes and tobacco cigarettes (18), consistent with the former study (47). However, the authors also found that sustained electronic cigarette use predicted an increase in self-reported depression symptoms at 12 months, thereby revealing a bidirectional relationship between depressive symptoms and electronic cigarettes (18). As these two studies suggest differences in directionality between depression and electronic cigarette use, it is important to consider factors mitigating these conclusions. For instance, differences in age, education, and geographic location have all been shown to affect nicotine use (4), and thus, the reported findings may be interdependent on other sociodemographic or developmental factors. In addition, the dose of nicotine contained in the electronic cigarettes or amount consumed by the participants was not recorded or standardized, which may have confounded the correlation with depressive symptoms. Finally, self-reported symptoms are subjective and may not meet the criteria for major depressive disorder (51).

Often closely associated with depression, electronic cigarette use is also positively correlated with both self-reported anxiety symptoms and generalized anxiety disorder (GAD) in humans (17, 52–56). While the directionality of this association is unclear, like depression, the relationship between anxiety and electronic cigarette use may also be bidirectional. Evidence demonstrates that people with a greater score as assessed with the GAD survey are more likely to initiate future electronic cigarette use (17). Further, adolescent populations self-report vaping for relaxation and stress and anxiety coping (54). Conversely, electronic cigarette use may increase the risk of anxiety disorders, including phobias, obsessive–compulsive disorder, or a panic disorder (57). Among adolescents and young adults, electronic cigarette use increased the likelihood of anxiety-related disorders by 37% (57). In addition to electronic cigarette initiation and continued use, increased anxiety is also evidenced during nicotine withdrawal (58). Interestingly, individuals that self-report greater anxiety sensitivity experience greater barriers for cessation (56) and individuals with depression diagnoses are more likely to experience more severe withdrawal symptoms (59). All in all, these findings suggest an intertwined relationship between electronic cigarette use, anxiety, and depression, which put those already experiencing these disorders at greater risk for electronic cigarette use, difficulties in cessation, and increased withdrawal symptomology.

## Studying drug exposure in animal models

Preclinical research in animal models, particularly rodents, allows researchers to investigate the effects of drugs and chemical constituents in a controlled setting. A wide range of techniques are available to study drug exposure, including both passive and self-administration methods. Passive (a.k.a., experimenter-administered) exposure allows for the control of both dose and time of administration relative to other outcome measures (e.g., examination of brain activation after a discrete time period) (60). This method, however, eliminates the motivational aspects of dependence. In contrast, intravenous or vapor self-administration protocols allow for the examination of the motivational and reinforcing drug properties that lead to continued use and seeking behaviors, allowing researchers to investigate different aspects of addiction processes. Both intravenous nicotine and aerosolized nicotine self-administration assays have been established for both rats and mice (61–67). Intravenous nicotine self-administration is considered the most reliable and robust method to study nicotine dependence, craving, and withdrawal (68). Intravenous nicotine self-administration also allows for the precise quantification of the amount of nicotine infused by the animal (69), but there are translational limits with this approach as humans typically inhale most nicotine-containing products. Recently, vapor nicotine self-administration paradigms have been developed (61, 62, 70), in which animals inhale aerosolized nicotine to more closely mimic human nicotine consumption. However, measurement of the net amount of nicotine inhaled with vapor self-administration is not feasible; while blood samples can be used to examine nicotine and its metabolites, the time course of nicotine metabolism in rodents limits the accuracy of detection with exposure across a self-administration session duration (e.g., 1+ hour). However, electronic cigarette exposure can lead to clinically relevant pharmacokinetics that translate to human biology (71). To date, rodent vapor self-administration paradigms have been demonstrated to be less robust than intravenous self-administration; associations between drug reward and active versus inactive behavioral responding have been inconsistent across published experimental paradigms (62, 65). Nevertheless, both intravenous and vapor nicotine self-administration paradigms are valuable tools to investigate various aspects of nicotine dependence, and established protocols demonstrate blood levels of nicotine’s metabolites, cotinine, 5-hydroxycotinine and cotinine N-oxide, similar to that found in human smokers (60, 62, 65, 72–74).

Many rodent models have been used to assess a wide range of behaviors related to symptoms of psychiatric disorders (75). For instance, depression-like behaviors have been classically measured using behavioral despair tests, such as forced swim and tail suspension, where quicker immobilization times are inferred to be an indicator of behavioral despair (75). Anxiety-like behavior in rodents is often assessed using open field and elevated plus maze tests, in which more time spent in the periphery of open field or in the closed arms of the elevated plus maze are associated with anxiety-like effects (76). Behavioral assays can also be used to quantify motivational and consummatory behaviors, in which decreased reward consumption or motivation to obtain palatable food reward is considered an indicator of anhedonia (75). Intracranial self-stimulation (ICSS) can also serve as an assessment of anhedonia or aversive state, in which rodents in an aversive state have been found to press a lever or spin a wheel to obtain higher levels of brain electrical self-stimulation (77, 78). While these tools have proven useful in measuring depression- and anxiety-associated states in animals, translational limitations are present when trying to extrapolate to mental health disorders in humans, given complex cognitive and social factors contributing to the human psychological state. Therefore, behavioral assessments with rodent models can be used to examine the reinforcing, rewarding, and cognitive effects of a variety of constituents found in electronic cigarettes. For instance, data from rodents support findings from human studies associating electronic cigarette exposure with anxiety- and depression-associated behaviors (79–81). Specifically, withdrawal from nicotine was shown to induce persistent changes in anxiety-, depression- and compulsive-like behaviors following 7 weeks of electronic cigarette exposure (79, 80), and surprisingly, these behavioral changes persisted 90 days following the last electronic cigarette exposure in male mice (79). Behavioral changes have also been observed even in the absence of nicotine, as nicotine-free electronic cigarette vapor exposure was shown to result in anxiogenic phenotypes in both male and female mice (81). With these apparent behavioral changes associated with electronic cigarette exposure, additional animal model research studies are needed to specifically elucidate the neurobiological mechanisms driving, and resulting from, electronic cigarette use. In the following sections, we will review the impact of various constituents on biological processes with data derived from both *in vitro* and *in vivo* studies.

## Health consequences and neurobiological effects of electronic cigarettes constituents

**Figure 1:**
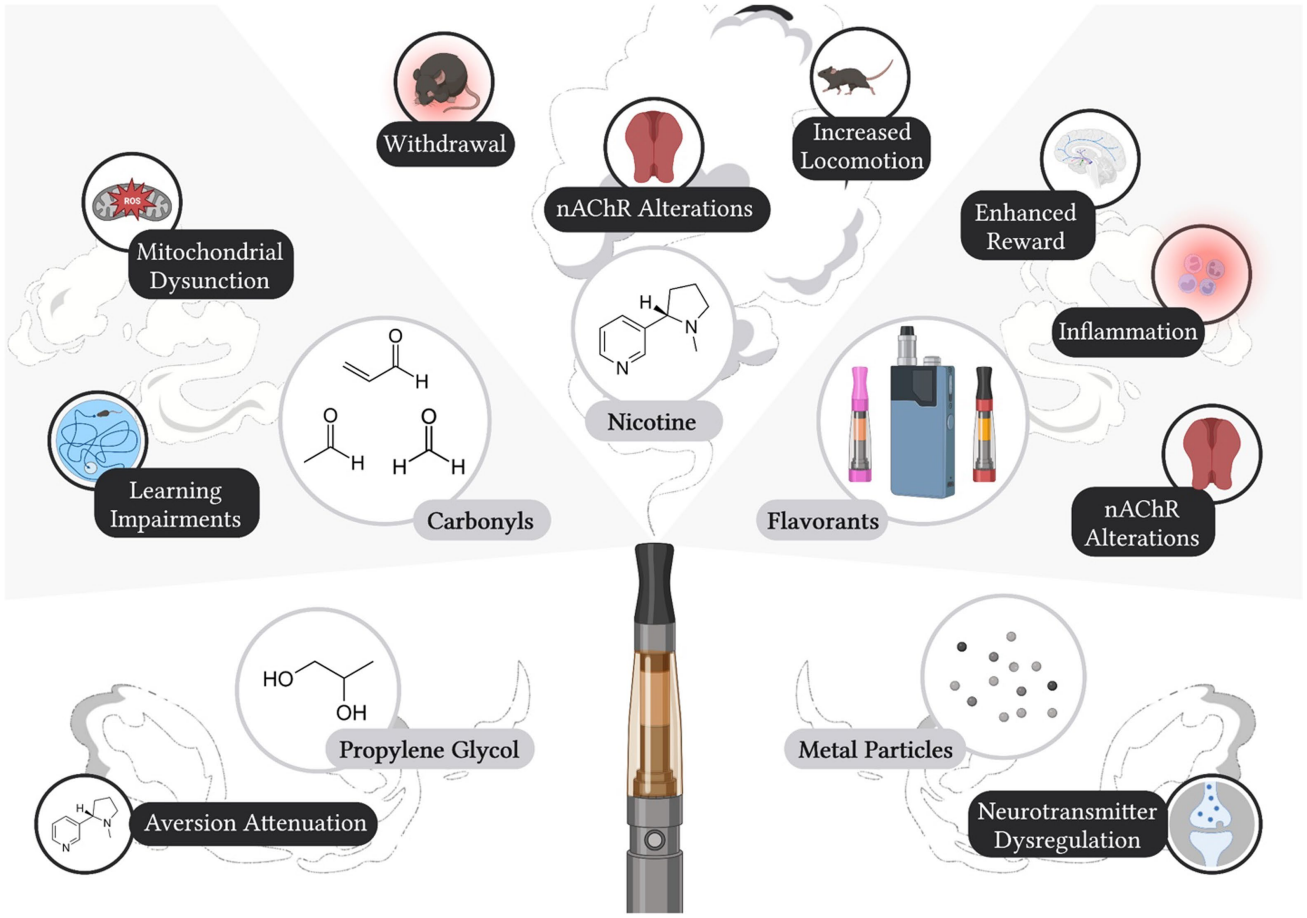
Labeled and Unlabeled Constituents in Electronic Cigarettes and Identified Effects on Neurobiology and Behavior. Electronic cigarette liquids and/or aerosols have been shown to contain nicotine, propylene glycol, carbonyls, flavorants, and metal particulates, all of which can induce effects on biological processes and/or behavior. Created with Biorender.com.

Electronic cigarettes have been promoted as a safer alternative to tobacco cigarettes, which has led to increased product use among various populations (25–28). However, electronic cigarette liquid and aerosols can pose many health risks to users (Figure 1). Further, while there is significant overlap in gene expression changes after electronic cigarette or tobacco cigarette use, there are several genes that are distinctly altered by electronic cigarette vapor and therefore may pose distinct health risks (82). Likely attributed to the lack of federal regulation, unlabeled components have been identified in electronic cigarette liquids with nuclear magnetic resonance spectroscopy analysis (83). Significant differences in labeled nicotine and actual nicotine content have also been found in commercially available electronic cigarette liquids, ranging from 35% less nicotine than what was labeled, up to 30% more nicotine than labeled (84). Alarmingly, nicotine has also been detected in electronic cigarette cartridges labeled as “nicotine-free” (84–86). These discrepancies between labeled and measured nicotine content in electronic cigarette liquids pose a significant risk to never smokers, who report a greater desire to use nicotine-free electronic cigarettes in comparison to nicotine-containing electronic cigarettes (87). Indeed, a study in Norway found that adolescents were three times more likely to vape nicotine-free electronic cigarettes as compared to nicotine-containing electronic cigarettes (88). While about half of nicotine-free electronic cigarette users from this cohort were able to quit the following year, it is important to note that from 2017 to 2019, about 15% of users each year transitioned from nicotine-free to nicotine-containing electronic cigarettes (88). Considering that nicotine has been detected in electronic cigarette liquids labeled as nicotine-free, these users may have unknowingly consumed nicotine, thereby priming their subsequent transition to nicotine-containing electronic cigarettes.

### Nicotine

Nicotine is the main psychoactive constituent present in both tobacco cigarettes and electronic cigarette liquid. Nicotine acts on ionotropic nicotinic acetylcholine receptors (nAChRs), where ligand binding results in channel pore opening and cation influx across the membrane (89). nAChRs are pentameric, assembling with various combinations of α and β nAChR subunits for a wide variety of nAChR subtypes with distinct pharmacokinetics, expression patterns, and actions on the cholinergic system (90, 91). Acting on nAChRs, nicotine exerts its reinforcing properties through the mesolimbic dopaminergic pathway (92). Systemic nicotine administration results in dopamine release in the nucleus accumbens (93), an effect shown to be mediated by β2* nAChRs in the ventral tegmental area (92, 94). Subsequent studies have demonstrated that the α4α6β2* nAChRs are necessary for nicotine self-administration in rodents (94). Moreover, nicotine also exerts aversive properties, particularly though the medial habenula to interpeduncular nucleus pathway (95). The medial habenula is enriched in α5*, α3*, β4* nAChRs, which have been shown to mediate nicotine aversion, drug-taking behavior, and/or withdrawal (96–101).

### Nicotine vape solutions

Propylene glycol and vegetable glycerin are additives commonly used as humectants (e.g., to control moisture levels in food and beauty products) and are the main liquids used to dissolve nicotine into solution to generate electronic cigarette aerosol for inhalation. Electronic cigarette solutions are marked commercially as “nicotine juice,” “e-juice,” “nicotine liquid,” or “nicotine solution.” Although the Food and Drug Administration recognizes propylene glycol and vegetable glycerin as “generally safe” for oral consumption, they have not yet been approved for inhalation (102). With electronic cigarette development and use significantly increasing within the last decade (4), the potential long-term effects of inhaled propylene glycol, vegetable glycerin, or both together on the brain and body have yet to be fully elucidated. Although propylene glycol and vegetable glycerin are both used in the electronic cigarette solutions, studies have focused on propylene glycol or the combined effects of vegetable glycerin and propylene glycol, rather than investigating vegetable glycerin alone.

Propylene glycol has been shown to interact with other compounds present in electronic cigarettes to induce synergistic effects. For example, HEK-293T cells exposed to both propylene glycol and vanilla electronic cigarette flavorant exhibited increased calcium signaling, which was attributed to activation of the aldehyde-sensitive receptor TRPA1 (103). Specifically, at higher concentrations, vanilla flavorant and propylene glycol together induced more robust calcium influx than either alone (103). TRPA1 receptors are expressed in brain microvasculature and play a functional role to maintain blood–brain-barrier integrity (104). Since acute exposure to non-flavored electronic cigarette aerosol was also shown to activate TRPA1 receptors in endothelial cells, this suggests that combined effects of propylene glycol and vanilla chemical flavorant may potentiate the adverse effect of each other or may exert greater side effects *in vivo* (105). Moreover, propylene glycol has also been shown to interact with nicotine to affect brain reward thresholds in male rats, as assessed with ICSS (78). While propylene glycol alone did not alter ICSS thresholds, co-administration of propylene glycol and nicotine decreased the aversion-associated increase in ICSS thresholds, which was induced by the high dose of nicotine alone (78). These findings indicate that at doses found in commercial electronic cigarette liquids, propylene glycol mitigates nicotine’s aversive properties and thus may promote higher levels of nicotine consumption (78), thereby increasing the product’s addiction liability. Maternal electronic cigarette exposure has also been shown to affect offspring. Surprisingly, prenatal exposure to vegetable glycerin with propylene glycol vapor was sufficient to induce deficits in long-term novel object memory (106), thus highlighting the importance of understanding the impact of electronic cigarette exposure *in utero.*

### Carbonyls

When heated together, propylene glycol and vegetable glycerin decompose to generate carbonyls in the aerosol, which most notably include acrolein, acetaldehyde, and formaldehyde (107–109). Many studies have found detectable levels of carbonyls in electronic cigarette aerosol, but variable levels have been reported across these studies, likely due to differences in the vape solution’s pH, heating temperature, propylene glycol/vegetable glycerin ratios, and individual differences in user vaping behavior (109–112). Importantly, in humans, these carbonyls can be detected in the airway following electronic cigarette use (113), and their respective metabolites can be further detected in the urine (108, 114, 115). While it is important to acknowledge that electronic cigarettes emit significantly fewer carbonyls than tobacco cigarettes (116), limited studies have investigated the level of carbonyl emission from electronic cigarettes, nor have they investigated the implications with long-term exposure.

Both acetaldehyde and acrolein are considered neurotoxins due to their effects on oxidative stress, which has been proposed to underlie neurodegenerative diseases including Alzheimer’s and Parkinson’s (117–119). Acetaldehyde and acrolein have been detected in both nicotine-free and nicotine-containing electronic cigarette aerosol, but interestingly, they are often not detected in the liquids themselves (86, 109, 120). These findings indicate that the process of heating the chemicals in the electronic cigarette liquid causes the formation of the aerosolized carbonyls. Metabolites of acetaldehyde and acrolein can also be detected in the urine following electronic cigarette vapor exposure (108, 115). Interestingly, both acetaldehyde and acrolein similarly impair cellular respiration. Acetaldehyde was shown to alter cellular respiration in cultured microvascular endothelial hBMVEC cells, which mainly comprise the blood–brain-barrier, thereby suggesting negative effects on blood–brain-barrier integrity (121).Further, in cultured primary cortical neurons, acetaldehyde impaired mitochondrial respiration via *NOX*-mediated activation (122), and in brain mitochondria *in vitro*, acrolein inhibited mitochondrial respiration via complex I (123). Similar effects have also been observed *in vivo,* as 3 days of electronic cigarette vapor exposure resulted in *NOX2-*mediated changes in mitochondrial respiration in the frontal cortex of male mice (124). In this study, acrolein was proposed to induce the *NOX2*-mediated impairments in cellular respiration (124). Interestingly, a study in humans revealed that current electronic cigarette use, but not past electronic cigarette use, was significantly correlated with mitochondrial DNA damage and dysfunction (82), which supports the translational relevance of these pre-clinical findings. Together, these findings indicate the acute nature of electronic cigarette exposure in mitigating negative outcomes.

In addition to acetaldehyde and acrolein, formaldehyde is also a product of propylene glycol and vegetable glycerin degradation (107–109). Formaldehyde is a well-documented carcinogen associated with adverse health consequences following acute exposure at higher doses, in addition to chronic lower levels of exposure (125). Formaldehyde levels in electronic cigarette aerosol have been documented to range from 0.07–0.15 parts per million (ppm), which is below the 5 ppm threshold for acute toxicity (126). Nevertheless, evidence demonstrates that chronic exposure to even low levels of formaldehyde can induce significant behavioral and molecular changes. For example, 7 days of gaseous exposure to a low dose of formaldehyde impaired spatial learning in the Morris water maze in male mice (127, 128). Furthermore, a chronically administered low dose of formaldehyde altered monoamine levels, including norepinephrine, epinephrine, dopamine, and serotonin, in the brain of male mice (127). Specifically, following 7 days of formaldehyde exposure at levels much lower than those emitted from electronic cigarettes, 0.0005 ppm formaldehyde exposure decreased norepinephrine and epinephrine levels, whereas 0.003 ppm decreased all of the monoamine levels (127). Moreover, 12 weeks of exposure of a low dose of aerosolized formaldehyde induced an upregulation in the number of corticotropin releasing hormone immunoreactive neurons in the paraventricular nucleus of female mice (129). Interestingly, an increased number of corticotropin releasing hormone neurons in the paraventricular nucleus has been observed in individuals diagnosed with major depressive disorder, as compared to healthy controls (130), suggesting clinical relevance of the findings from mice. Taken together, these studies provide evidence that long-term formaldehyde exposure at levels similar to electronic cigarette emission can lead to significant changes in brain mechanisms underlying cognitive function, potentially including depression.

### Metals

Electronic cigarettes have been considered ‘safer’ than combustible tobacco cigarettes, such as with lower carbonyl levels. However, a main counterindication of this ‘safe’ assessment is the presence of inhaled metal particulates in electronic cigarette aerosols, which are at levels greater than that found in tobacco cigarette smoke (131, 132). Metals are likely leached into electronic cigarette liquids and vape during aerosol production, when electronic cigarette liquids come into contact with the metal heating coil (133). Like carbonyls, these metals can also be detected in the urine, saliva, exhaled breath, and blood of electronic cigarette users (131, 133). Electronic cigarette-emitted metal particulates have been shown to readily cross the blood–brain-barrier, as evidenced by metal accumulation in the mouse brain (134). Moreover, following electronic cigarette exposure, arsenic, chromium, copper, iron, manganese, nickel, lead, selenium, strontium, and zinc were found to accumulate across different regions of the brain, with the greatest accumulation observed in the anterior frontal cortex and striatum (134). Specifically, in the anterior frontal cortex, copper and strontium were enriched, whereas arsenic, chromium, copper, iron, lead, and selenium were enriched in the striatum (134). Like other types of electronic cigarette emissions, the type and relative proportion of metals that might accumulate in the brain are expected to vary among commercially available brands due to differences in the vape liquid constituents and electronic cigarette device characteristics (135). Of note, metal inhalation has been shown to induce behavioral changes in male rats, including a decrease in locomotor activity following chromium inhalation (136). Both arsenic and lead are well-known neurotoxins, with the greatest negative effects occurring during earlier neurodevelopmental stages. Maternal exposure to either arsenic or lead during gestation or developmental exposure during adolescence has been shown to result in an overall increase in monoamine signaling in the brain, which persisted into adulthood in rats (137). Unexpectedly, co-exposure of both arsenic and lead led to an opposing effect with decreased monoamine signaling (137). These data highlight the potential differential effects of each constituent and unknown effects with multiple metals present in the aerosols. This illustrates the need to expand our understanding of the effects of metals in electronic cigarettes for various stages of neurodevelopment. It is also important to note that metal components are often found in higher concentrations in less expensive products (24, 138–140), which would presumably be purchased at higher levels by those of lower socioeconomic status or adolescent users with limited income. Given this, future research should be directed at investigating whether increased health disparities will become more evident with long-term product use by marginalized communities of lower socioeconomic status.

### Electronic cigarette flavorants

As the electronic cigarette market constantly evolves, more palatable flavorants consistently emerge to attract a broader audience of consumers. In 2014, a study identified over 7,000 commercially available electronic cigarette flavors (141), which does not take into account the many variations of chemicals used to create a singular flavor (e.g., fruit or candy). Mint and fruit electronic cigarette flavors are preferred by individuals across ages (young adult to adult) and smoking status (never, current, or former electronic cigarette/tobacco cigarette user) (142–144). Thus, an enhanced understanding of how the chemicals used to generate the variety of electronic cigarette flavorants impact the reinforcing properties of nicotine, drug use patterns, and health outcomes will be essential to ascertain. However, this has been difficult for the scientific community given the numerous chemical combinations used to generate the flavors and limited time/resources as commercialized flavors constantly evolve from company to company and across time.

#### Mentholated products

One of the most consistent findings for the impact of flavorants on nicotine product use is the effect of menthol. More positive attitudes have been reported toward mint/menthol flavored electronic cigarettes compared to tobacco flavored electronic cigarettes, which was evidenced by an increased reported satisfaction and increased likelihood to repeat use (145). In those who smoke mentholated tobacco cigarettes, decreased smoking cessation rates are found (146), indicating that mentholated products have increased addiction liability. Unfortunately, due to focused marketing by companies, individuals that are younger and/or from marginalized ethnic backgrounds disproportionately use menthol-containing products (146–149), which has led to notable health disparities within our society. Findings in humans have been supported by rodent models that demonstrate menthol enhances the rewarding and reinforcing properties of nicotine. For instance, administration of menthol-flavored electronic cigarette extract reduced a conditioned taste aversion, as compared to nicotine alone, in a two-bottle test with male and female adolescent rats (150). Following menthol administration, male rats self-administered more intravenous nicotine infusions and increased their motivation to obtain nicotine (151), and male mice self-administered electronic cigarette vapor containing menthol and nicotine at greater levels than nicotine vapor alone (61). Together, these findings provide strong evidence that the addition of menthol to electronic cigarette products leads to greater dependence and addiction liability.

#### Green apple flavored products

Green apple is a characterizing fruit flavor commonly found in electronic cigarette liquids, which is acquired by the addition of the chemical farnesol (61, 152). In adolescents, the green apple flavorant was found to increase vaping behavior compared to both menthol-flavored and unflavored electronic cigarettes (152). These findings in humans may be due to a positive association of green apple based on the individual’s history (e.g., positive conditioning with fruit candy consumption as a child) and/or due to biological effects of the chemical on the reward-related neurocircuitry of the brain. Henderson and colleagues have led a series of important studies that reveal the biological effects of farnesol. They found that green apple-flavored nicotine vapor is self-administered at a higher level than unflavored nicotine vapor in adult male mice (61), supporting the enhanced reinforcing properties of farnesol with nicotine. Further, green apple flavorant alone can induce a conditioned place preference, in addition to enhancing nicotine’s rewarding effects, in both male and female adult mice (153, 154), thereby demonstrating rewarding properties on its own. At the cellular level, farnesol can affect the kinetics of the nicotinic acetylcholine receptor, which is the receptor on which nicotine binds to induce its reinforcing and rewarding effects (4, 153). Specifically, 24 h of farnesol pretreatment induced a shorter desensitization period for the nicotinic receptors containing the α4 and β2 subunits (153), which would allow for more permissible receptor re-activation in the presence of nicotine. Chronic farnesol administration also increased the firing rate of nicotinic acetylcholine receptor-expressing neurons in the ventral tegmental area (153). Thus, the addition of the green apple flavorant induces neurobiological changes in the brain’s reward-related circuitry, which enhances nicotine’s rewarding properties to reinforce continued product use.

#### Impact on inflammatory processes

Nicotine has been shown to induce both inflammatory and anti-inflammatory effects based on a number of factors, which may include dose, duration of treatment, route of administration, and underlying mechanisms (67, 155, 156). In general, nicotine has been characterized as exerting mainly anti-inflammatory effects throughout the brain and body (157). Like tobacco cigarette smoke, flavored electronic cigarette vapor has also been shown to induce pro-inflammatory markers in the brain, potentially due to nicotine, other constituents present in the vapor, or the interaction of nicotine and the constituents (158, 159). After 14 days, grape flavored electronic cigarette exposure increased in TNF-α in the cerebral cortex of male mice (158). A common electronic cigarette brand, JUUL, provides the vape liquid in an encapsulated pod, and vapor emitted from the JUUL pod has been shown to induce several pro-inflammatory responses in nucleus accumbens sub-regions in female mice following long term exposure (159). In the nucleus accumbens shell, aerosol from both mint/menthol and mango flavored JUULs increased the expression of TNF-α, IL-1β, and IL-6 following 1 and 3 months of exposure (159). In contrast, inflammatory markers in the nucleus accumbens core increased in a time-dependent manner; TNF-α expression was increased following both 1 and 3 months of JUUL exposure, but IL-1β was increased only following 1 month of JUUL exposure (159). Consistent with nicotine’s anti-inflammatory effects, chronic nicotine administration via an osmotic minipump did not increase either TNF-α or IL-1β in the nucleus accumbens in male mice (160), thereby supporting the notion that the constituents in the JUUL pods, or the interaction of nicotine with the constituents, led to the changes in inflammatory markers. Thus, these findings highlight the need to understand the potential impact of different chemical constituents on signaling in the brain.

In summary, based on the findings reviewed above, it is evident that all components of electronic cigarette emissions, including those labeled and unlabeled, can possess the potential to alter reward-related processing and behavior. However, it is important to acknowledge that the relative amounts of acrolein, acetaldehyde, formaldehyde, and metals released from electronic cigarette aerosol vary and may be lower than that examined in these reviewed studies. It is equally important to acknowledge that drug use also affects individuals on a longitudinal scale, and as such, chronic exposure to different constituents present in electronic cigarette aerosol may influence health outcomes, which will not be revealed until after many years of product use. Moreover, individual constituents may combine to induce synergistic effects that are different than each constituent alone, as evidenced by propylene glycol exposure and metal particulate exposure. Given that electronic cigarette use has been associated with cognitive effects, such as depression (18), and common biological pathways metabolize nicotine and psychiatric medications, it is also important to consider the intersection of electronic cigarette constituents and metabolizing enzymes.

## Alterations to CYP450 enzyme function and drug interactions

Cytochrome P450 (CYP450) enzymes play a critical role in drug metabolism, especially for therapeutic compounds used to treat symptoms associated with depression and other psychiatric disorders. Tobacco cigarette smoking has been shown to affect the expression of different enzymes within the CYP family, thus increasing the risk for CYP-mediated drug interactions. Most notably, polycyclic hydrocarbons in tobacco cigarette smoke induce expression of CYP1A2, which is essential for the breakdown of the antidepressant fluvoxamine (161); thus, given the increased metabolism, one would expect a reduced effect of fluvoxamine in a chronic tobacco smoker. However, the current clinical implications of electronic cigarette use and prescription drug interactions are largely unknown. In this section, we will review current scientific data derived from pre-clinical studies.

*In vitro* studies have demonstrated that electronic cigarette liquid can alter CYP450 enzyme activity in various cell culture conditions (162–164) (Table 1). Administration of vape liquid has been shown to upregulate the expression of CYP2A6, CYP2U1, CYP2E1, and CYP2S1 mRNA (162), and exposure to condensed electronic cigarette aerosol induces CYP1A1 and CYP1B1 activity (164). In contrast, solution from nicotine-free vape liquids (strawberry poptart and apple watermelon flavors) was shown to inhibit the activity of CYP2A6 (163).Together, these findings demonstrate both CYP isoform-specific and direction-specific effects following exposure to different electronic cigarette solutions. Of note, CYP2A6 is the isoform that metabolizes nicotine (73, 174), indicating a potential for altered drug use patterns based on the constituents in the vape liquid.

**Table 1:** CYP450 alterations *in vitro* or *in vivo* following exposure to constituents in electronic cigarettes.

*In vitro* evidence					
Cytochrome	Constituent	Cell culture	Direction		Reference
CYP1A1	Condensed electronic cigarette aerosol	MSK Leuk1	Increase		(164)
CYP1A2	Polycyclic hydrocarbons	HepG2; MCF-7	Increase		(165)
CYP1B1	Condensed electronic cigarette aerosol	MSK Leuk1	Increase		(164)
CYP2A6	Electronic cigarette liquid	hCMEC/D3	Increase		(162)
CYP2A6	Nicotine-free electronic cigarette flavors -strawberry poptart, apple watermelon	Microsomal Recombinant CYP2A6	Decrease		(163)
CYP2E1	Electronic cigarette liquid	hCMEC/D3	Increase		(162)
CYP2S1	Electronic cigarette liquid	hCMEC/D3	Increase		(162)
CYP2U1	Electronic cigarette liquid	hCMEC/D3	Increase		(162)
*In vivo* evidence					
Cytochrome	Constituent	Animal, Sex	Direction	Substrate	Reference
CYP1A2	Formaldehyde	Rat, Male	Increase	Phenacetin	(166)
CYP2B	Nicotine	Rat, Male	Increase	Propofol	(167)
CYP2C11	Formaldehyde	Rat, Male	Decrease	Testosterone	(166)
CYP2D6	Nicotine	African Green Monkey Rat, Male	Increase	Desipramine Doxepin Imipramine Maprotiline Minanserin Nortriptyline Protriptyline Trimipramine Fluoxetine Codeine	(168–171)
CYP2E1	Propylene glycol formaldehyde	Mice, Male Rat, Male	Decrease	Chlorzoxazone	(166, 172)
CYP3A2	Formaldehyde	Rat, Male	Decrease	N/A	(166)
CYP450 (non-specific)	Acrolein	Rat, Male	Decrease	N/A	(173)

Panels highlight the CYP450 isoform affected, the electronic cigarette constituent examined, cell/animal model system, and the directionality of the effect on the CYP450 isoform. Data from in vivo findings also indicates pharmacological drug compounds that can be affected by changes in metabolism.

*In vivo* studies have provided further insight into the potential clinical implications associated with altered CYP450 metabolism. In an important study by Khokhar and Tyndale, the authors found that 7 days of nicotine treatment increased CYP2B expression in the brain, but not liver, and surprisingly, this change in metabolism was sufficient to potentiate the sleep-inducing effects of the general anesthetic propofol in male rats (167). In addition to nicotine, carbonyls may also influence metabolism. For instance, acrolein has been found to inhibit CYP450 enzymes (173), and formaldehyde specifically reduces the levels of CYP2C11, CYP2E1, and CYP3A2, but increases CYP1A2, in male rats (166). Interestingly, the effects of formaldehyde on enzymatic activity led to decreased testosterone (steroid hormone) and chlorzoxazone (muscle relaxant) clearance, but increased phenacetin (analgesic) clearance (166). Propylene glycol has further been shown to inhibit CYP2E1, leading to decreased chlorzoxazone clearance by greater than 80% in male mice (172). It is interesting to note that CYP2E1 is a minor metabolizer of some antidepressants, including the selective serotonin reuptake inhibitor, fluoxetine, and monoamine oxidase inhibitor, moclobemide (168).

The CYP2D enzyme family is a well-documented major metabolizer of antidepressants, including desipramine, doxepin, imipramine, maprotiline, mianserin, nortriptyline, protriptyline, and trimipramine (168). Interestingly, in the frontal cortex, hippocampus, striatum, and cerebellum, chronic nicotine administration was shown to upregulate CYP2D6 mRNA in male rats (169) and CYP2D mRNA and protein in African green monkeys (170). This nicotine-mediated CYP2D induction has been shown to be sufficient to alter codeine metabolism in the brain but not in the plasma of male rats (171). Furthermore, these alterations were enough to increase codeine’s analgesic effect during the tail flick test (171). This suggests that long-term electronic cigarette use may alter the metabolism of these antidepressants, thereby affecting therapeutic efficacy and side effects. Of further note, genetic polymorphisms have been associated with drug bioavailability. For instance, altered blood plasma bioavailability of the antidepressant fluoxetine is found in individuals expressing *CYP2D6* polymorphisms (175). Specifically, different allelic variations in *CYP2D6* influence enzyme activity leading to either ultra-metabolizers or poor-metabolizers of CYP2D6 substrates (175, 176). Theoretically, a higher level of metabolism would be expected to terminate the drug action sooner, thereby limiting the effectiveness of the drug. In contrast, a decreased metabolism would be expected to allow for prolonged drug effectiveness and/or to increase the likelihood of off-target adverse effects with increased side-effects due to accumulation of the drug compound. Indeed, individuals with genetic polymorphisms in *CYP2D6* were more likely to have participated in more antidepressant medication trials and also have been shown to switch among antidepressants more often (176, 177). These findings could either suggest that *CYP2D6* polymorphism leads to: (1) an increase in depression severity/incidence or (2) an insufficient therapeutic response and/or excessive side effects. Given that studies have failed to find an association between *CYP2D6* polymorphism and incidence of depression (178), the most likely conclusion is the latter, in which individuals have an increased need to try different therapeutics in search of a positive therapeutic response (176, 177). In sum, individual constituents found in electronic cigarette liquid and aerosol can significantly impact CYP450 enzyme activity (Table 1), which represents an important consideration when evaluating therapeutic effectiveness in the clinic for patients suffering from depression.

## Conclusion

The recent development and use of electronic cigarettes, as well as a general lack of regulatory oversight, has led many individuals to be exposed to chemicals that have unknown long-term effects on the brain. While a clear positive correlation between electronic cigarette use and depression has been established (18, 47), the causality and mitigating factors affecting this relationship are largely undetermined. Therefore, as we go forward, it will be necessary to more precisely investigate the acute and long-term effects of all constituents found in the aerosols and the impact of these factors at various stages of neurodevelopment. This includes both labeled ingredients, such as propylene glycol, vegetable glycerin, nicotine, and various chemical flavorants (menthol, green apple, mango, and others), as well as unlabeled ingredients present in the aerosols of the devices. These can include carbonyls (acetaldehyde, acrolein, formaldehyde) and heavy metals (arsenic, chromium, copper, iron, manganese, nickel, lead, selenium, strontium, zinc). It will also be important to consider the effects of these constituents on the CYP450 enzyme family and related implications for therapeutic efficacy of psychiatric medications. Due to the large variation in the quantities of each constituent across products and an ever-evolving product marketplace, it will continue to be challenging to fully understand the clinical relevance of electronic cigarette use on the individual’s health. However, given our current understanding and the potential adverse implications for public health, regulatory agencies should take a more proactive role in overseeing the production and commercialization of electronic cigarette products.

## Author contributions

MB, AM, and CDF contributed to the manuscript drafts, and all authors approved the final version. MB created the figure.

## Funding

This work was supported by grants from the Tobacco-Related Disease Research Program (TRDRP) (T31IR1767 and T32IR4866 to CDF and T32DT5202 to MB).

## Conflict of interest

The authors declare that the research was conducted in the absence of any commercial or financial relationships that could be construed as a potential conflict of interest.

## Publisher’s note

All claims expressed in this article are solely those of the authors and do not necessarily represent those of their affiliated organizations, or those of the publisher, the editors and the reviewers. Any product that may be evaluated in this article, or claim that may be made by its manufacturer, is not guaranteed or endorsed by the publisher.
